# Supplementary material for: Pro-Apoptotic Activity of MCL-1 Inhibitor in Trametinib-Resistant Melanoma Cells Depends on Their Phenotypes and Is Modulated by Reversible Alterations Induced by Trametinib Withdrawal
Source: Cancers (Basel). 2023 Sep 29;15(19):4799. doi: 10.3390/cancers15194799 (PMC10571954; doi:10.3390/cancers15194799)
Supplement: Supplementary file 1 [file cancers-15-04799-s001.zip › cancers-2597368-supplementary.pdf]

cancers

MDPI

Supplementary Material: Pro-Apoptotic Activity of MCL-1 Inhibitor in Trametinib-Resistant Melanoma Cells Depends on Their Phenotypes and Is Modulated by Reversible Alterations Induced by Trametinib Withdrawal

Mariusz L. Hartman, Paulina Koziej, Katarzyna Kluszczyńska and Małgorzata Czyz

Equation S1. The mathematical model with an efficiency correction used to calculate the relative level of each transcript by qRT-PCR [47].

$$relative\ transcript\ level = \frac{(E_{ref})^{CP\ sample}}{(E_{target})^{CP\ sample}} \div \frac{(E_{ref})^{CP\ calibrator}}{(E_{target})^{CP\ calibrator}}$$

E—qRT-PCR efficiency of reference (E_{ref}) gene (RPS17) or target (E_{target}) gene (MCL-1 and NOXA)

CP_{sample}—crossing point of the sample i.e., sample of interest (e.g., DH 1d, DH 8d, re-TRA 1d, etc.)

$CP_{calibrator}$—crossing point of the sample that was used as a reference i.e., 21_TRAR or 29_TRAR, sample for which transcript level was set as 1.

Table S1. Mutation status of genes encoding components of the core apoptotic machinery. Only non-synonymous mutations and indels are included. Mutations are marked as homozygous (+/+) or heterozygous (+/−). Prediction of functional effects of amino acid substitutions was assessed by using Polyphen-2 software, and was classified as benign (scores 0.000–0.449), possibly damaging (scores 0.450–0.959), and probably damaging (scores 0.960–1.000). The name of the protein is given in the brackets if it differs from the gene name.

Gene	DMBC21	21_TRAR	DMBC29	29_TRAR
APAF1	N782T +/−<br>benign 0.027	N782T +/−<br>benign 0.027	N782T +/−<br>benign 0.027	N782T +/−<br>benign 0.027
BAD				
BAK1 (BAK)				
BAX				
BBC3 (PUMA)				
BCL2				
BCL2A1	G82D +/−<br>possibly damaging 1.000<br>N39K +/−<br>benign 0.052<br>C19Y +/−<br>benign 0.000	G82D +/−<br>possibly damaging 1.000<br>N39K +/−<br>benign 0.052<br>C19Y +/−<br>benign 0.000	G82D +/−<br>possibly damaging 1.000<br>N39K +/−<br>benign 0.052<br>C19Y +/−<br>benign 0.000	G82D +/−<br>possibly damaging 1.000<br>N39K +/−<br>benign 0.052<br>C19Y +/−<br>benign 0.000
BCL2L1 (BCL-X$_L$)				
BCL2L2 (BCL-w)	Q133R +/+<br>benign 0.000	Q133R +/+<br>benign 0.000	Q133R +/+<br>benign 0.000	Q133R +/+<br>benign 0.000
BCL2L11 (BIM)				
BECN1				
BID				
BIK				
BIRC2 (cIAP1)				
BIRC3 (cIAP2)				
BIRC5 (survivin)	E152K +/+	E152K +/+	E152K +/+	E152K +/+

	benign 0.000	benign 0.000	benign 0.000	benign 0.000
BIRC7 (livin)				
BMF				
BNIP3L				
CASP3				
CASP7	D340E +/−<br>benign 0.000	D340E +/−<br>benign 0.000	D340E +/−<br>benign 0.000	D340E +/−<br>benign 0.000
CASP8	K14R +/−<br>benign 0.008	K14R +/−<br>benign 0.008	K14R +/−<br>benign 0.008	K14R +/−<br>benign 0.008
CASP9	A28V +/−<br>possibly damaging 0.793<br>Q221R +/−<br>benign 0.000	A28V +/−<br>possibly damaging 0.793<br>Q221R +/−<br>benign 0.000	A28V +/−<br>possibly damaging 0.793<br>Q221R +/−<br>benign 0.000	A28V +/−<br>possibly damaging 0.793<br>Q221R +/−<br>benign 0.000
CFLAR (FLIP)				
CYCS<br>(cytochrome c)				
DIABLO				
FADD				
FAS				
FASLG				
HRK				
MCL1				
PMAIP1 (NOXA)				
TNF				
TNFRSF1A (TNFR1)				
TNFRSF10A (TRAILR1)	R441K +/+<br>benign 0.000	R441K +/+<br>benign 0.000	R441K +/+<br>benign 0.000	R441K +/+<br>benign 0.000
TNFRSF10B (TRAILR2)	V191A +/-<br>benign 0.001	V191A +/+<br>benign 0.001	V191A +/+<br>benign 0.001	V191A +/+<br>benign 0.001
TNFRSF21 (DR6)				
TNFSF10 (TRAIL)				
TRADD				
TRAF1	P46L +/−<br>benign 0.000	P46L +/−<br>benign 0.000	P46L +/−<br>benign 0.000	P46L +/−<br>benign 0.000
TRAF2				
XIAP	Q423P +/−<br>benign 0.002	Q423P +/+<br>benign 0.002	Q423P +/+<br>benign 0.002	Q423P +/+<br>benign 0.002

Corresponding to Figure 1B

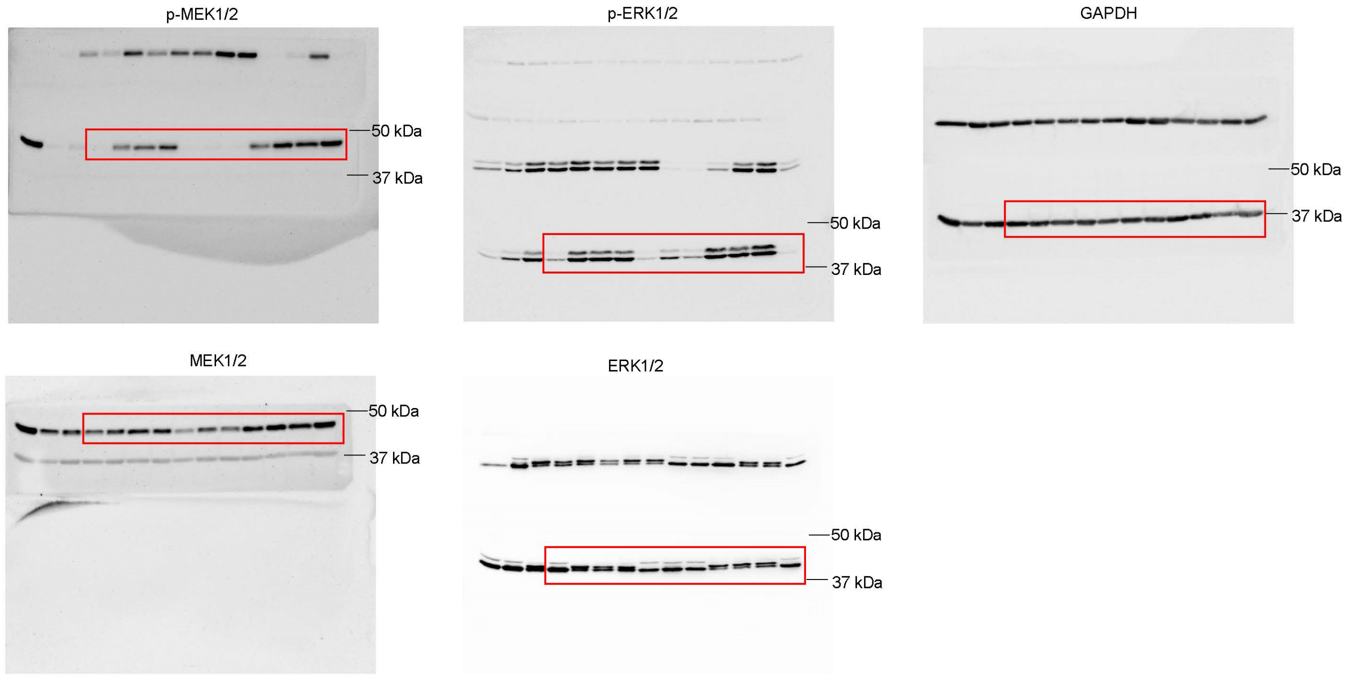

Corresponding to Figure 1C

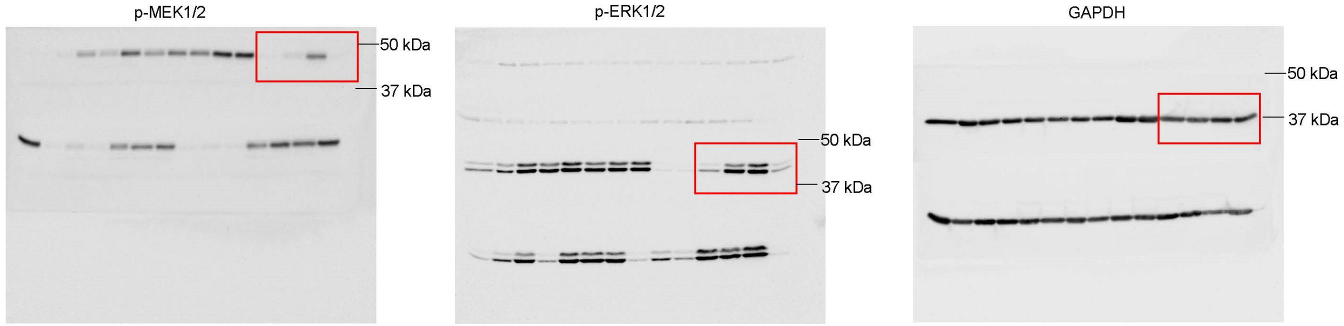

Corresponding to Figure 1D

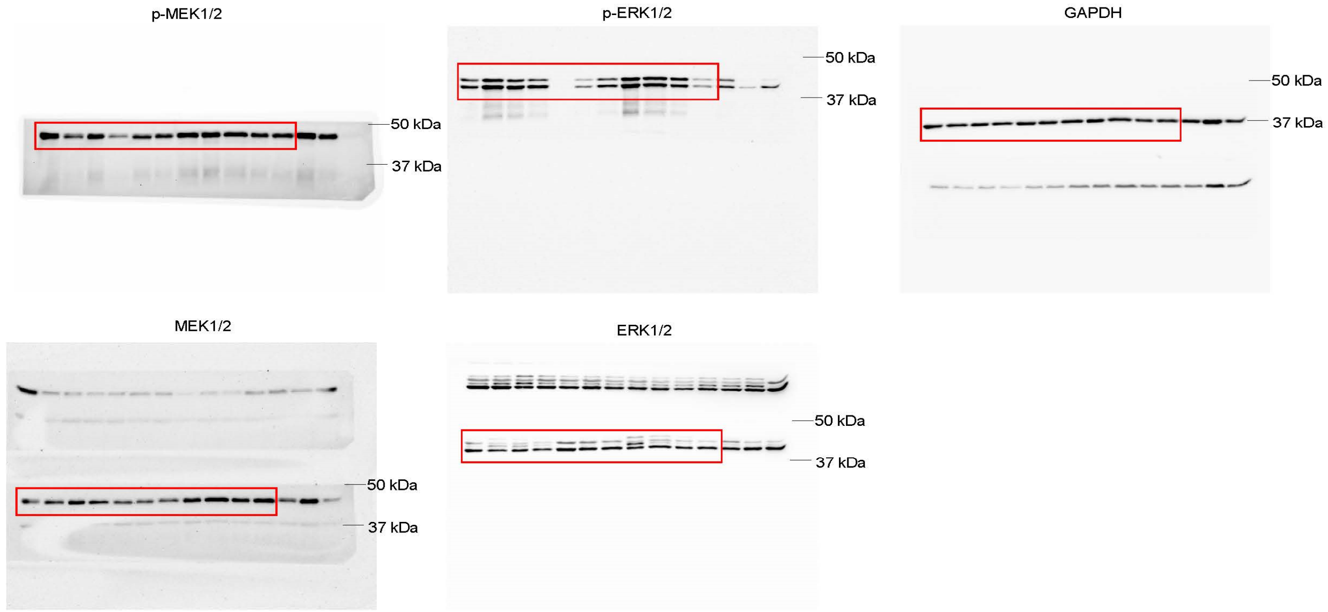

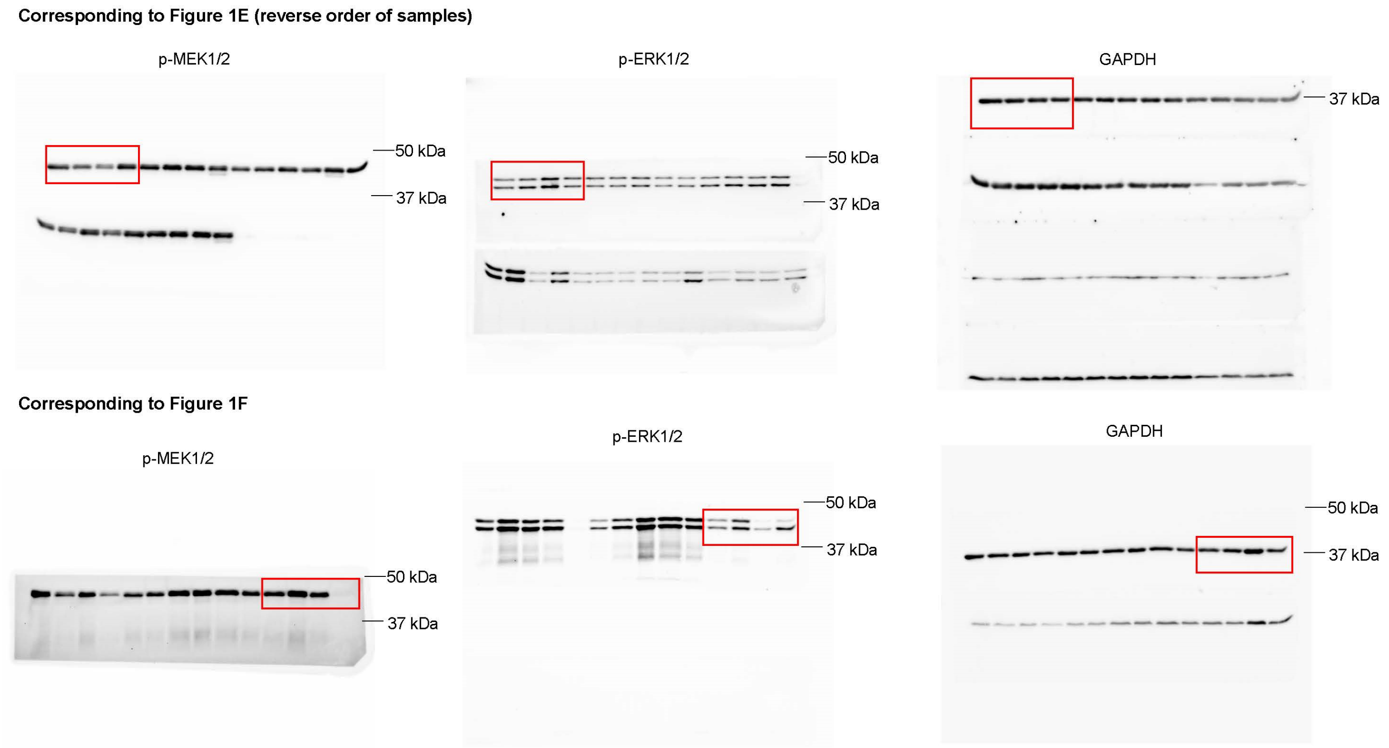

Figure S1. Uncropped Western Blot images for Figure 1.

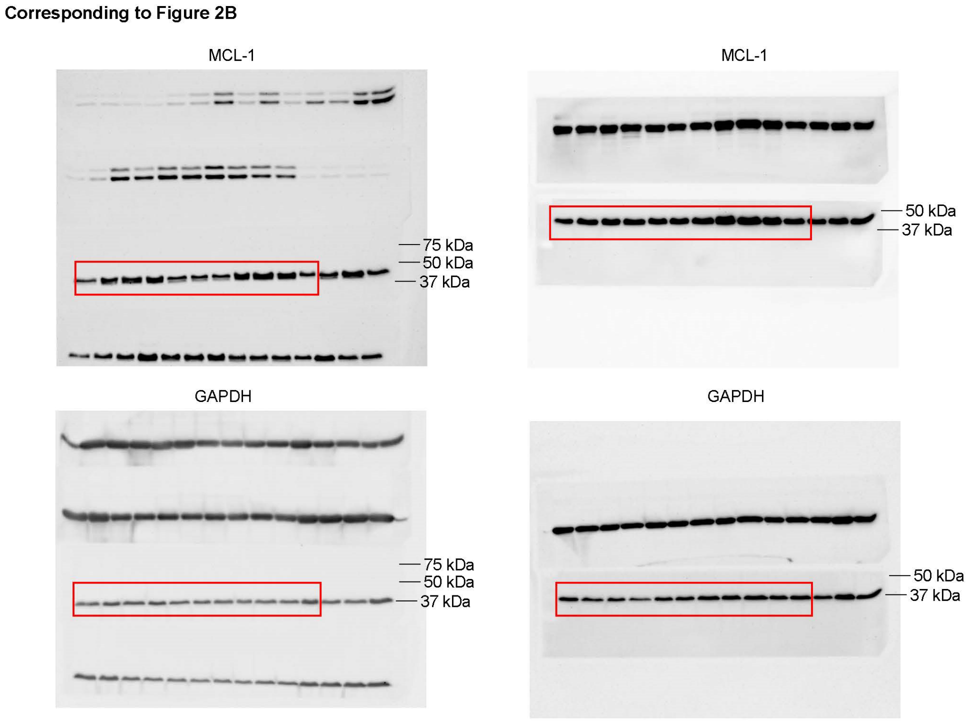

Corresponding to Figure 2D (left panel)

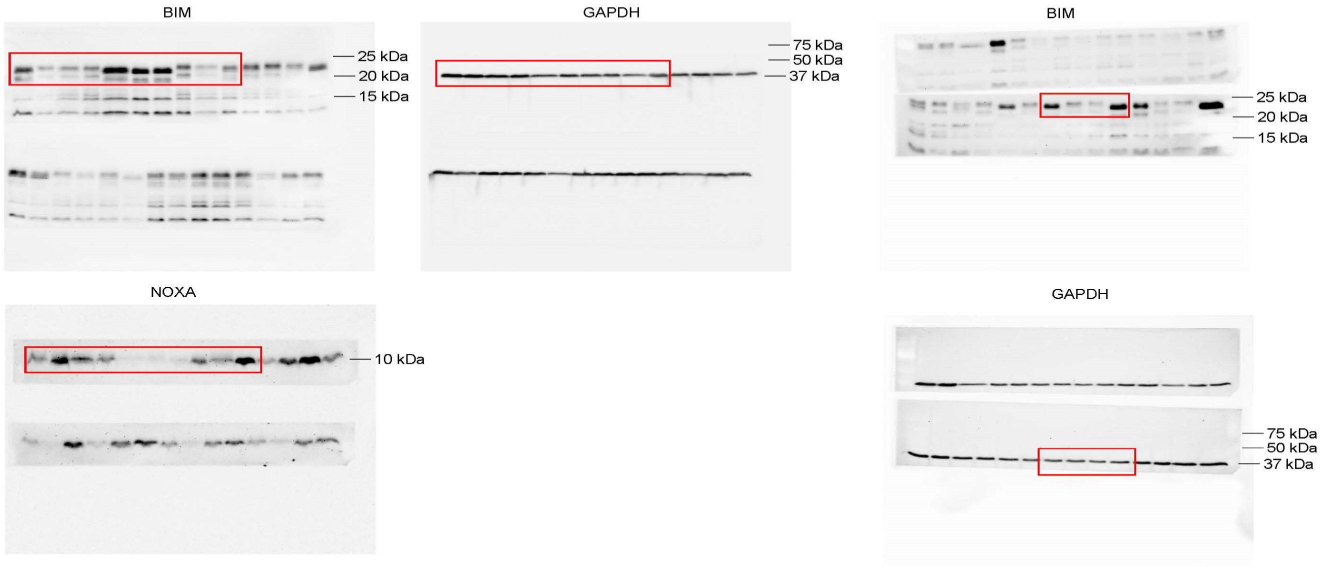

Corresponding to Figure 2D (right panel)

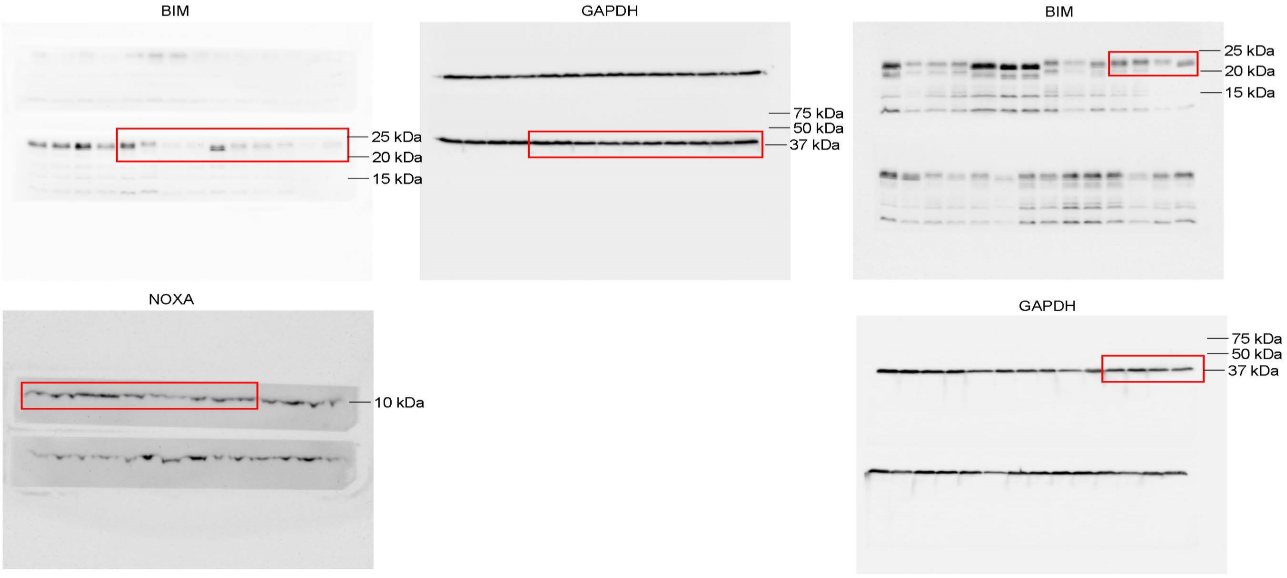

Corresponding to Figure 2E (left panel)

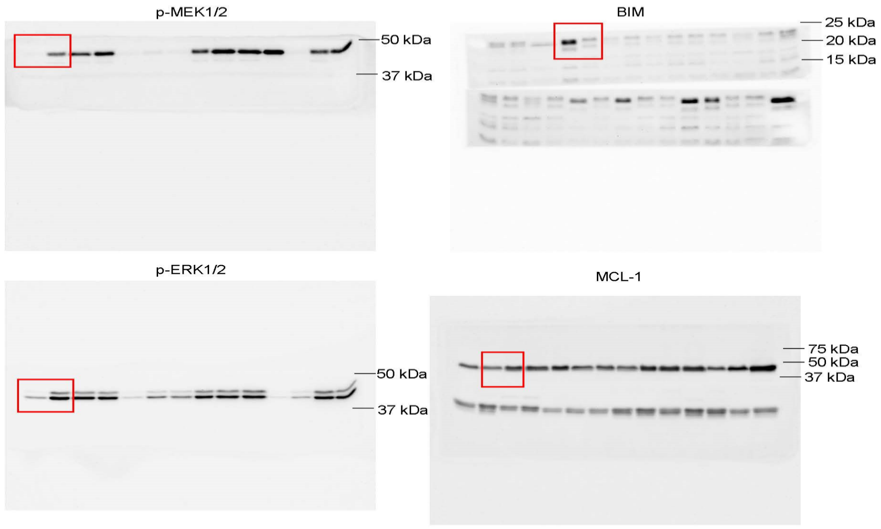

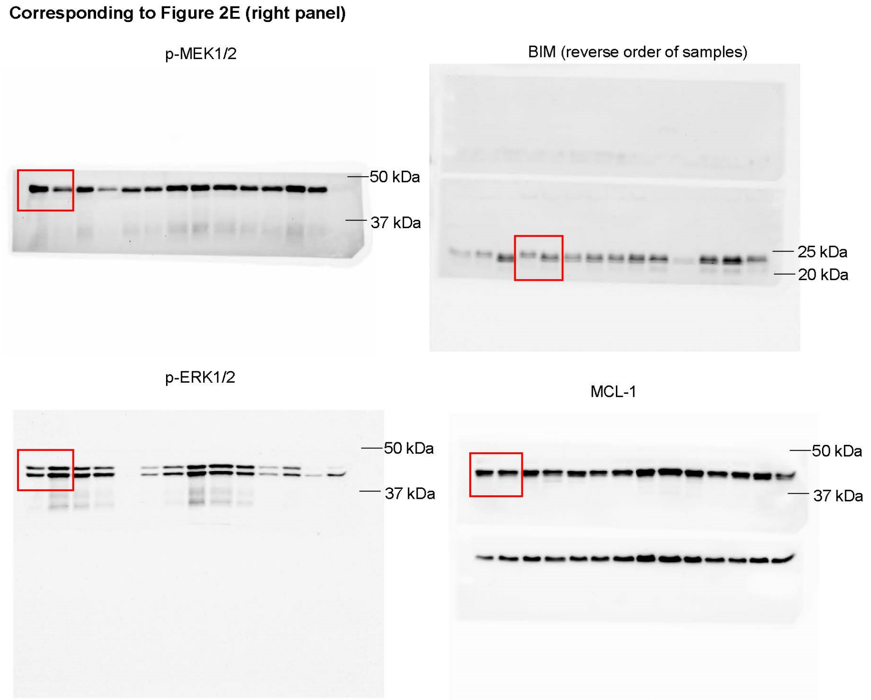

Figure S2. Uncropped Western Blot images for Figure 2.

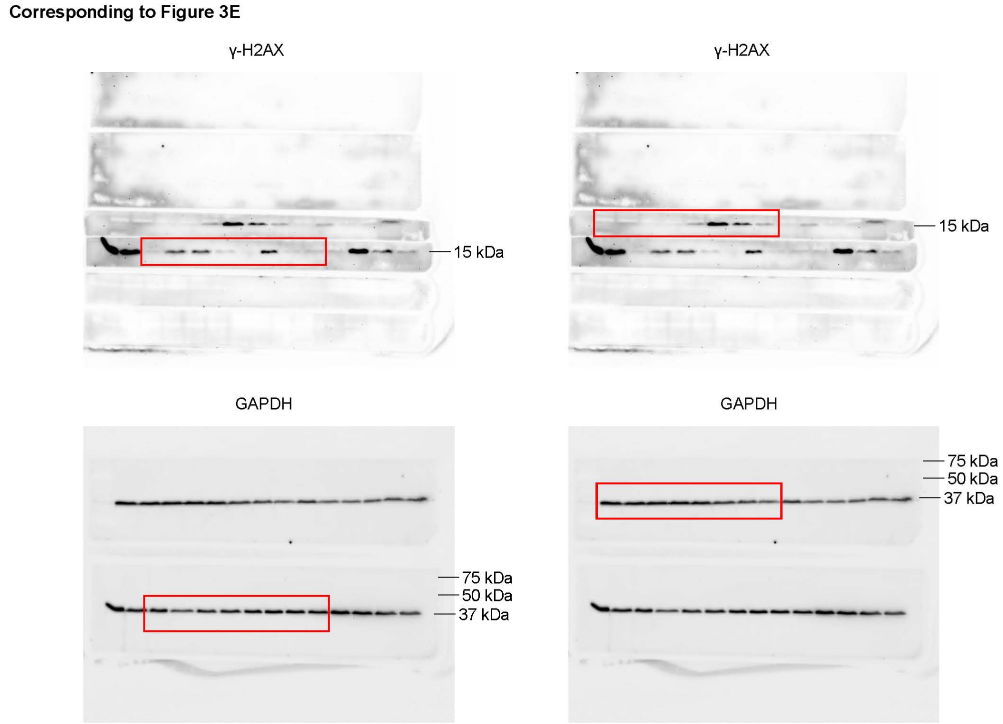

Figure S3. Uncropped Western Blot images for Figure 3.

Corresponding to Figure 4A

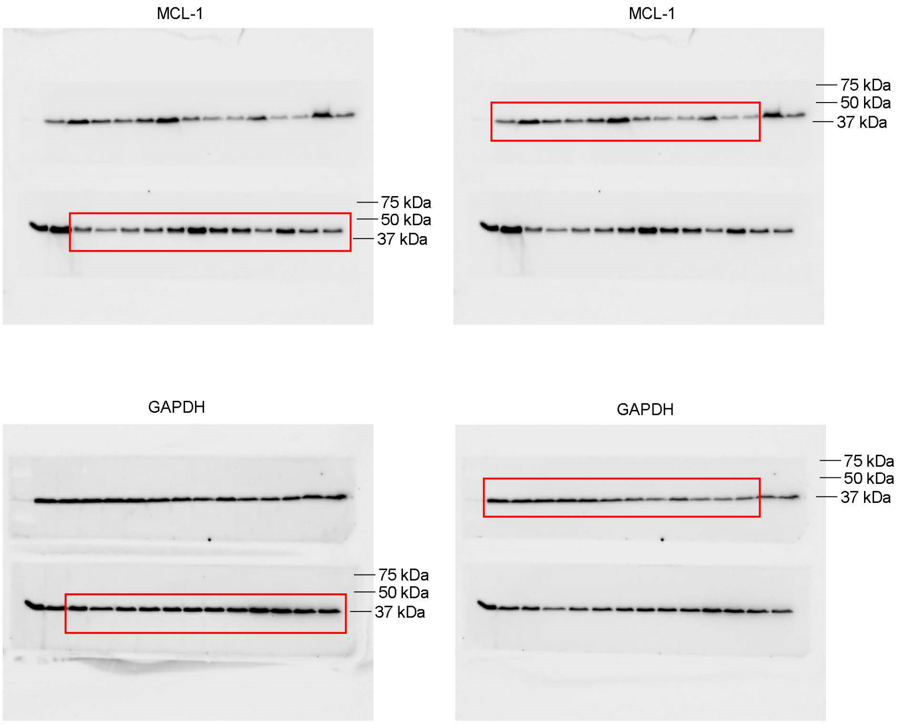

Corresponding to Figure 4C

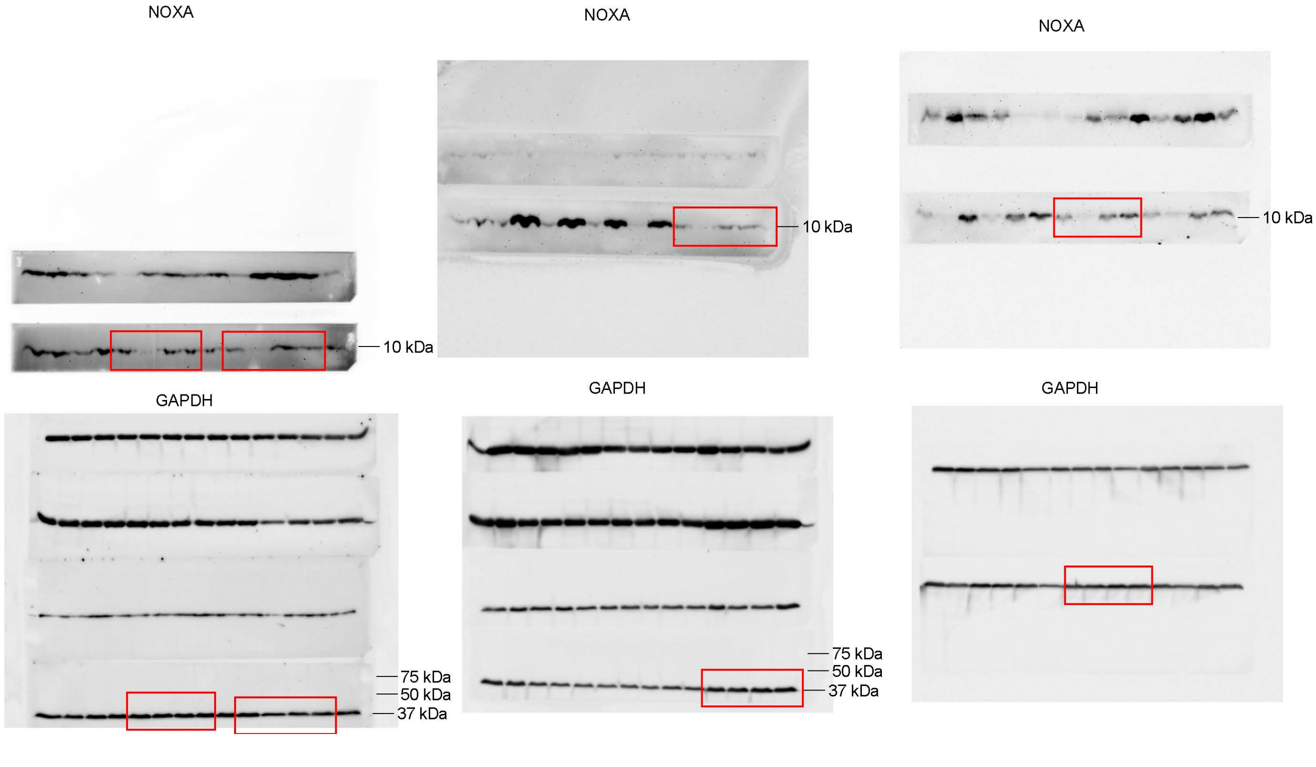

Corresponding to Figure 4D (left panel)

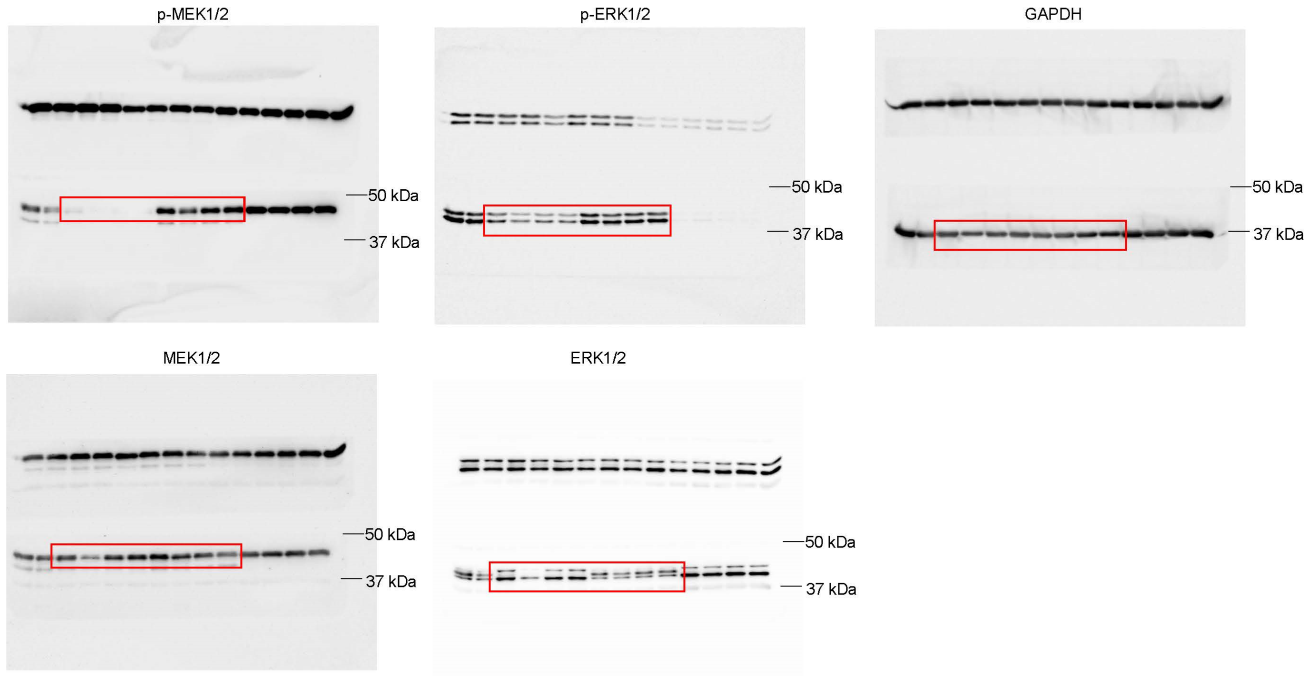

Corresponding to Figure 4D (right panel)

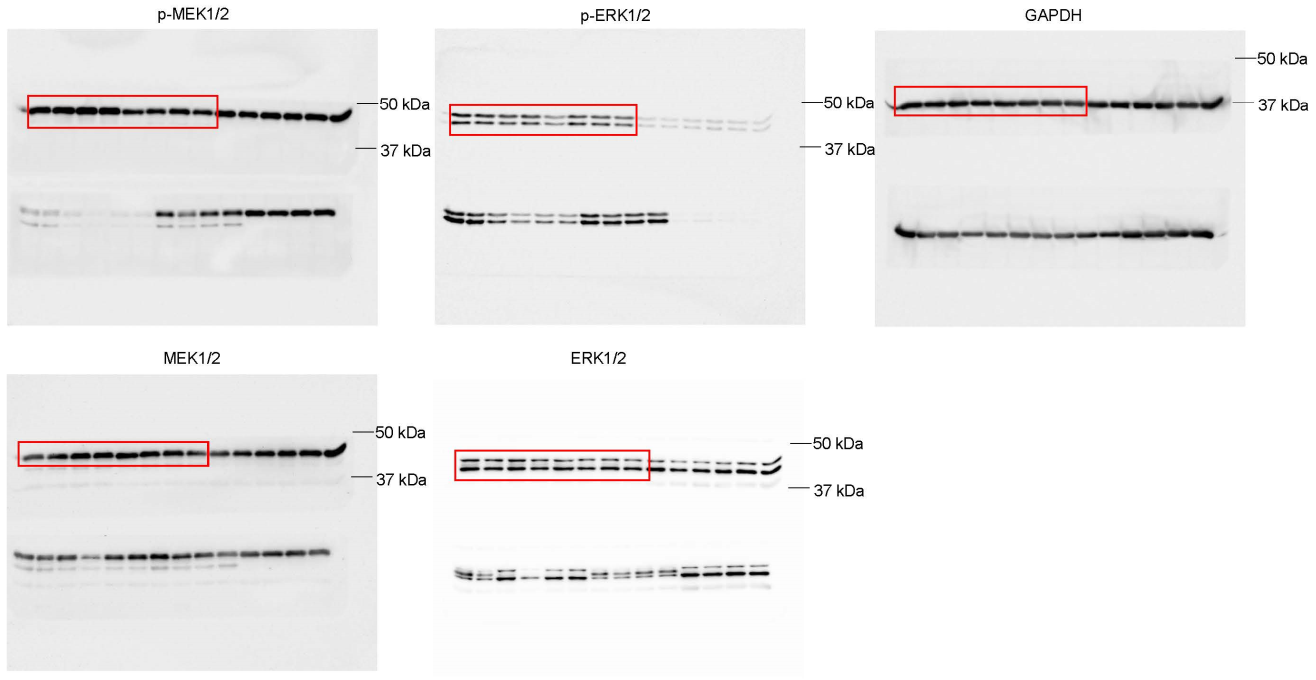

Figure S4. Uncropped Western Blot images for Figure 4.
